# Effect of a proprietary intraluminal stiffening wire device on cecal intubation time and rate with used colonoscopes; a randomized, controlled trial

**DOI:** 10.1186/1756-0500-6-48

**Published:** 2013-02-04

**Authors:** Jeffrey M East

**Affiliations:** 1 Department of Surgery, Cornwall Regional Hospital (CRH), Montego Bay, Jamaica; 2 Department of Surgery, Radiology, Anesthesia and Intensive Care, University of the West Indies, Mona, Kingston 6, Jamaica

**Keywords:** Used colonoscope, Old colonoscope, Colonoscope stiffness, Colonoscope stiffening device, Colonoscope stiffening wire

## Abstract

**Background:**

Colonoscopes are designed with balance between flexibility, required to negotiate angulations, and stiffness, required to counteract the propensity for looping in unfixed sections of the colon, which retards advancement of the instrument. Colonoscopy can be challenging with old instruments that have lost native stiffness and become less responsive to torquing.

A new intraluminal stiffening device has become available in two grades of stiffness. However, there is no published evidence of its effectiveness. This randomized, controlled trial was designed to determine the effectiveness of the stiffening wires in improving cecal intubation rate and time following routine application. A secondary analysis determines effectiveness of application only after intractable failure with the unaided colonoscope.

**Methods:**

The colonoscope tested was an Olympus CF-100TL, approximately fifteen years old. Patients were randomly assigned to the unaided colonoscope or the standard or firm wire introduced routinely on entry into transverse colon. Each phase of colonoscopy was timed. Failure to advance the colonoscope for 5 minutes (despite usual manipulations to minimize looping) required switching to another intervention according to a prescribed methodology and the originally assigned intervention was recorded as failed.

**Results:**

The study was terminated after accrual of 112 participants (target sample size 480) because the colonoscope required repairs (no damage attributable to stiffening wires) which would have been uneconomical. There were no statistically significant differences between per-protocol cecal intubation rates (81.1, 71.1 and 70.3 percent respectively), a finding which persisted after multiple imputation for a virtual sample size of 480. Similarly, there were no statistically significant differences between per-protocol cecal intubation times (15, 16.2 and 13.9 minutes). However, a statistically significant improvement in cecal intubation rate (from 81.1% to 97.3%, P = 0.0313) was achieved when the wires were applied after intractable failure of the unaided colonoscope in the first intervention group.

**Conclusions:**

Routine application of either stiffening wire does not improve caecal intubation rate nor time compared to the unaided colonoscope. However, application of the stiffening wires after intractable failure of the unaided colonoscope enabled a statistically significant improvement in cecal intubation rate.

**Trial Registration:**

clinicaltrials.gov Identifier: NCT01115010

## Background

Colonoscopes are designed with a delicate balance between flexibility, required to negotiate angulations, and stiffness, required to resist the tendency for looping to occur in the unfixed sections of the colon, a phenomenon which retards advancement of the instrument [1,2]. An increase over the native stiffness of traditional, fixed-stiffness colonoscopes is achieved by torquing the shaft or application of a stiffening device [3,4]. Both native stiffness and responsiveness to torquing decrease in colonoscopes that have been in use for a long time [4,5] and although this can be restored by replacing the insertion tube, that would increase the cost of used colonoscopes significantly. Overtubes seem to have fallen out of favour in recent times, for reasons which are unclear from the literature, but probably include fear of perforation and the fact that they are cumbersome to apply [3]. Intraluminal stiffening wires enjoyed a brief run but were also abandoned because of their potential to cause damage to the instrument channel [3,6].

Most colonoscopies in developing countries are performed with old, used colonoscopes purchased from developed countries, because patients cannot afford to pay the level of fees required to offset the cost of new equipment. Colonoscopy with used, excessively flexible instruments can be challenging [4].

A relatively cheap, FDA approved, proprietary intraluminal stiffening wire device has become available in 2 grades of stiffness [7]. There is, however, no research evidence in the literature that the stiffening wires actually improve the efficiency or effectiveness of colonoscopy and, if they do, whether maximum benefit is achieved by routinely introducing the wires at the first opportunity allowed by the manufacturers (on entry into the transverse colon) or only after failed advancement of the unaided scope.

A randomized, controlled trial was therefore designed to test the effect of routine application of both grades of the stiffening wire device on caecal intubation rate and time. The effect of application of the wires after intractable failure of the unaided colonoscope is also tested. Statistical analysis in the first case is by bivariate hypothesis testing using chi-squared test and *t*-test as appropriate, and in the second, by the exact McNemar’s test for paired data from small samples.

## Methods

The research protocol was approved by the separate ethics committees of the Western Regional Health Authority, St. James, Jamaica, and of the University of the West Indies at Mona, Kingston, Jamaica. Informed consent for the research (and colonoscopy) was received for all participants. All colonoscopies included in the study were performed by the author using a single, pre-owned, adult colonoscope, an Olympus CF-100TL, manufactured in the early 1990s (and therefore approximately 15 years old at start of study).

The stiffening wires are made by Zutron Medical™ [7] and both grades of stiffness (ZUTR-141700 (Standard)™ and ZUTR-161700 (Firm)™) were tested in this study. The wires have a flexible, rounded tip ostensibly to avoid transmission of stiffness to the bending section and minimize the risk of channel damage. The manufacturers recommend that the device be introduced through the suction/biopsy channel of colonoscopes with working length not less than 168 cm only after the colonoscope has entered the transverse colon and any loop formed in the sigmoid colon has been reduced. Eight centimetres of heat shrink tubing was applied just beyond the handle of each device to improve the grip of the device by the biopsy channel valve and prevent leakage of insufflated air, a problem identified during pre-study usage of the wires.

All patients 18 years old and over presenting for diagnostic or screening colonoscopy were eligible for inclusion. Females post hysterectomy and males post radical prostatectomy (previous pelvic surgery is associated with difficult colonoscopy [8]), patients with prior colon resection and patients with a history suggestive of prior incomplete bowel obstruction or in whom a barium enema showed a stenosing lesion likely to obstruct passage of the colonoscope were excluded.

For 80% power to detect a completion rate increase from 85% to 95% as significant at the 5% level, number of participants needed is 160/group (Epi Info Version 3.5.1). For 80% power to detect a difference in mean time from splenic flexure to hepatic flexure (or hepatic flexure to cecum) of 3.5 mins. (assuming a mean of 7 mins. for controls, a mean of 3.5 mins for cases (reduction by 1/2), a range of 1.34 – 10 and therefore a standard deviation of 1.44 [(10–1.34)/6] as significant at the 5% level, number needed is 132/group (PS Power and Sample Size Calculator, Version 2.1.31 [9]). Target sample size was set at the higher number of 480 cases (160/group).

The randomization schedule was prepared prior to the start of the study. The blocked randomization technique was used, to ensure equal numbers in each intervention category, with a block size of 9 and with numbers selected from a random numbers table [10]). Allocation of intervention to patients was made, based on the printed randomization schedule which was retrieved from the drawer in which it was sequestered, in the order in which they had the procedure performed and only after inclusionary and exclusionary criteria were satisfied, consent to the procedure and the research were received and the patient positioned on the table.

Interventions were as follows:

A. Colonoscopy without stiffening wire.

B. Standard wire introduced on entry into transverse colon.

C. Firm wire introduced on entry into transverse colon.

Each phase of colonoscopy was timed with a stop watch with a “split lap” function (Nike Triax 50™). Phases timed were: from insertion into anus to entry into transverse colon; from entry into transverse colon to entry into ascending colon; from entry into ascending colon to intubation of cecum. Time required for passage and withdrawal of the wires was excluded. If, at any stage after entry into the transverse colon, the tip of the colonoscope fails to progress for a period of 5 minutes (despite all the usual manipulations to prevent and undo looping, including pull-back, application of abdominal pressure and change of position), switching to another intervention is required and the originally assigned intervention is considered to have failed. Switching procedures for each intervention are as follows:

A. On failure (as defined above) of the unaided colonoscope in group A, the standard wire is introduced. If the standard wire fails, it is withdrawn and the firm wire is introduced. If the firm wire fails, it is withdrawn and the procedure continues with the unaided colonoscope. This cycle of effectively varying the stiffness of the colonoscopy is continued in this order until the cecum is successfully intubated or the procedure abandoned.

B. On failure of the standard wire in group B, the next option is to withdraw the wire and proceed, followed by the firm wire if the unaided colonoscope fails, and so on, until the cecum is successfully intubated or the procedure abandoned.

C. On failure of the firm wire in group C, the next option is to withdraw the wire and proceed, followed by the standard wire if the unaided colonoscope fails, and so on, until the cecum is successfully intubated or the procedure abandoned.

All patients received 20 mg of hyoscine intravenously and were sedated by titration with intravenous meperidine and midazolam. Stiffening wires were lubricated prior to passage in order to reduce shearing stress between the wire and the wall of the biopsy channel.

Variables recorded for each patient were age, sex, previous abdominal surgery, quality of bowel preparation, endoscopy assistant (there were two), intervention, time to entry into transverse colon, time from entry into transverse colon to entry into ascending colon, time from entry into ascending colon to cecal intubation, switch from assigned intervention required or not and, if switch required, in what part of the colon, whether cecal intubation successful by assigned intervention or not, and if not, whether cecal intubation eventually achieved or not. Data were recorded unto a pre-coded data collection form and then entered into a STATA Version 11 database (copy of dataset referenced here [11]) for statistical analysis.

Bivariate analyses were performed to determine whether any recorded variables, such as age, sex, quality of bowel preparation and endoscopy assistant were potential confounders for the effect of the stiffening wires on either of the two major outcome variables (time to cecum and cecal intubation rate) and to test the integrity of the randomization process. The main analysis consisted of bivariate testing, by chi-squared test or *t*-test as appropriate, for differences in effect on the main outcome variables (cecal intubation rate and time) between intervention groups. Within group A, a determination was made, using the exact McNemar’s test for paired data, of the statistical significance of any improvement in cecal intubation rate attributable to introduction of the wires after intractable failure of the unaided colonoscope.

## Results

After accrual of 112 subjects, the colonoscope required repairs which were estimated to cost more than the cost of a refurbished, later model replacement. Since the study is colonoscope specific, it was terminated. The repair technicians have confirmed that there was no damage to the wall of the biopsy channel from application of the stiffening wires.

Thirty seven patients were randomized to procedure A, 38 to procedure B and 37 to procedure C. Two cases allocated to procedure C were excluded from the per-protocol analysis as in both colonoscopy was terminated before the assigned intervention was introduced. One was the only case of perforation occurring during the study and in the other, colonoscopy was terminated because the patient became uncooperative. The perforation occurred at the recto-sigmoid junction in an 84 year old man. There were no complications attributable to use of the wires.

Mean age, gender, quality of bowel preparation and endoscopy assistant (all known or plausible risk factors for difficult colonoscopy [12,13]) were not associated with either caecal intubation rate or time by bivariate and crude logistic regression modelling, confirming that the randomization process was effective in equitably distributing these potential confounders (and likely unmeasured confounders as well, such as body mass index [12,13], variability of operator performance over time, etc.) and justifying the simple bivariate models used for the main comparisons which follow.

Table 1 displays cecal intubation rates successfully achieved without having to switch from the protocol-assigned intervention (hereafter referred to as per-protocol rates) and total cecal intubation rate in each intervention group (which includes those cases in which the cecum was successfully intubated after failure of the protocol-assigned intervention and hereafter referred to as intention-to-treat rates). The results of bivariate statistical comparisons are displayed in the legend. Table 2 displays corresponding cecal intubation times.

**Table 1:** Per-protocol and intention-to-treat cecal intubation rates by intervention

	**Intervention**		
	**Unaided colonoscope (A)**	**Standard wire (B)**	**Firm wire (C)**
Number	**37**	**38**	**37**
Per-protocol Cecal intubation rate	**(30) 81.1%** (CI, 67.8 – 94.3%)	**(27) 71.1%** (CI, 55.9 – 86.2%)	*(26/35) 74.3% (CI, 59.1 – 90%)
Intention-to-treat Cecal intubation rate	**(36) 97.3%** (CI, 91.8 – 100%)	**(35) 92.1%** (CI, 83.1 – 100%)	**(31) 83.8%** (CI, 71.3 – 96.2%)

*Two cases in this group are excluded from the per-protocol analysis as failure occurred before the assigned intervention could be introduced.

Chi-squared test of difference between per-protocol A and B cecal intubation rate, P = 0.31.

Chi-squared test of difference between per-protocol A and C cecal intubation rate, P = 0.49.

Exact McNemar’s test of difference between intention-to-treat and per-protocol cecal intubation rate under intervention A, P = 0.0313.

**Table 2:** Per-protocol and intention-to-treat cecal intubation times by intervention

	**Per-protocol intervention**		
	**Unaided colonoscope (A)**	**Standard wire (B)**	**Firm wire (C)**
Per-protocol Cecal intubation time	**15** mins. (CI, 13.6 – 16.4; range, 8 – 25.7)	**16.2** mins. (CI, 14.2 – 18.2; range, 8.4 – 26.2)	*13.9 mins. (CI, 12–15.8; range, 6.8 – 23.6)
Intention-to-treat Cecal intubation time	**16.7** mins. (CI, 14.9 – 18.5; range, 8 – 32.5)	**18.1** mins. (CI, 15.6 – 20.6; range, 8.4 – 40)	**15.4** mins. (CI, 13.3 – 17.4; range, 6.8 – 26.1)

*Two cases in this group are excluded from the per-protocol analysis as failure occurred before the assigned intervention could be introduced.

*t*-test of difference between per-protocol A and B cecal intubation time, P = 0.32.

*t*-test of difference between per-protocol A and C cecal intubation time, P = 0.31.

It is the per-protocol analyses that address the comparative efficacy of the different interventions. Intention-to-treat rates and times are reported in the tables but not the results of intention-to-treat comparisons as it is not effectiveness that is being compared. In any case, it is reasonable to expect that intention-to-treat rates and times would be the same over time in each intervention group since, after failure of the allotted intervention, the procedure (switching between all 3 interventions as required) is essentially the same for each group.

Overall caecal intubation rate was 91.1% (CI; 85.7-96.4%). The per-protocol caecal intubation rate for procedure A was not significantly different from that for procedure B (P = 0.31, chi-squared test) nor procedure C (P = 0.49, chi-squared test) (Table 1). Analyses after multiple imputations for a virtual sample size of 478, in which data from unaccrued cases are treated as missing values [14], also yield insignificant P-values for the per-protocol rate comparison between procedures A and B (P = 0.31, logistic regression) and between procedures A and C (P = 0.49, logistic regression).

The wires significantly improved cecal intubation rate (at the 5% level of significance) after failure of the unaided colonoscope, in patients assigned to Procedure A, from 81.1% to 97.3% (P = 0.031, exact McNemar’s test). There is no evidence of greater effectiveness of one wire versus the other in achieving this improved cecal intubation rate, as the increase is equally distributed between the two grades.

All per-protocol failures under Procedure A (that is, failures of the unaided colonoscope), in cases in which caecal intubation was eventually successful, occurred in the ascending colon (6/36, 16.7%) whereas Procedures B and C failed in the transverse colon in 4/35 (11.4%) and 2/31 (6.5%) respectively and in the ascending colon in 4/35 (11.4%) and 3/31 (9.7%) respectively. In other words, the standard and firm wires reduced the likelihood of negotiating the hepatic flexure by 11.4% (CI–1.8-26.8%) and 6.5% (CI; -3.6-20.3%) respectively in cases randomized to procedures B and C in whom the caecum was successfully intubated, whereas no per-protocol failures among cases randomized to procedure A occurred in the transverse colon.

Overall mean cecal intubation time was 16.8 minutes (CI; 15.5-18; range, 6.8–40). The mean per-protocol cecal intubation time for procedure A was not significantly different from that for procedure B (P = 0.32, *t*-test) nor procedure C (P = 0.31, *t*-test). Analyses after multiple imputations for a virtual sample size of 478 (as performed above for cecal intubation rate comparisons) also yield insignificant P-values for the per-protocol comparisons of cecal intubation time for procedure A versus B (P = 0.32, linear regression) and procedure A versus C (P = 0.31, linear regression).

## Discussion

This study has not identified any benefit, by way of higher cecal intubation rate nor reduced cecal intubation time, from routine introduction of the stiffening wires on entry into the transverse colon compared with use of the unaided colonoscope. However, application of the stiffening wires after intractable failure to progress with the unaided colonoscope enabled a statistically significant increase in cecal intubation rate over the rate achieved with the unaided colonoscope.

No studies were identified in the literature in which intraluminal stiffening wires were tested for efficacy and effectiveness. Parallels therefore have to be drawn from studies in which the new, variable stiffness colonoscopes (VSC), designed to deliver increased stiffness on demand [1,15], are compared to traditional colonoscopes (albeit of more modern vintage than the one tested in this study) or in which the performance of the VSC is compared at different stiffness settings.

In a meta-analysis of seven randomized controlled trials comparing VSCs with regular adult colonoscopes, Othman et al. [16] reported that VSC use was associated with a marginally higher cecal intubation rate, but only among less experienced colonoscopists, and that cecal intubation times were similar for the two colonoscope types. Hsieh et al. [17] reported results of a randomized study with three arms, in which the variable stiffness function of a VSC was activated only after intractable failure to progress in the baseline arm (“no activation”), routinely after reaching the descending colon in the second arm (“regular”) and on an “as needed” basis in the third arm. There was no statistically significant difference in cecal intubation rate nor time for the per-protocol analysis between the three arms. In the “no activation” group, 100% cecal intubation was achieved after the stiffness function was activated in the two cases in which there was intractable failure with the “no activation” setting. The finding in both studies, that routine application of the increased-stiffness settings of the VSC has no clinically significant advantage over a traditional adult colonoscope or a VSC with unactivated variable stiffness function, parallels the similar finding in this study in relation to routine application of the stiffening wires. The finding in the study by Hsieh et al., that caecal intubation rate improved on application of the variable stiffness function after failure with the VSC in unactivated mode, also parallels the similar, secondary finding in this study in relation to application of the stiffening wires after intractable failure of the unaided colonoscope.

Are the stiffening wires likely to be helpful with newer (traditional) colonoscopes than the one used in this study? Since the cecal intubation rate achievable with an unaided newer colonoscope is expected to be higher than that achieved with the old colonoscope used in this study, it is unlikely that any increase resulting from application of the stiffening wires after intractable failure of a newer colonoscope is going to be statistically significant. However, any increase at all is clinically important and cost effective (given the relatively low cost of the wires).

If increased stiffness reduces or prevents the tendency for looping to occur, which is known to be the major reason for difficulty in advancing the colonoscope, why should the routine application of increased stiffness not result in improved cecal intubation rate as well as time? Failure to improve cecal intubation rate in the per-protocol analysis is likely due to failure to completely undo looping before application of the wires or to recurrence of looping. Failure to improve cecal intubation time after routine application of the stiffening wire may be due to the possibility that temporal gains from reduced looping in the sigmoid colon are being offset by difficulty negotiating the hepatic flexure. Both stiffening wires appear to inhibit successful negotiation of the hepatic flexure in some cases (see Results above) whereas there were no failures proximal to the hepatic flexure with the unaided colonoscope among cases in which the cecum was successfully intubated. Ginsberg [1] points out that “floppy …scopes…may better traverse excessively angulated sections” and Hsieh et al. [17] noted that in two of their cases assigned to “regular” activation of the variable stiffness function, “the VSF had to be released because the scope was so rigid that it could not pass a tortuous segment”.

Early termination of the study, before accrual of the target sample size, was unavoidable as continuation with a different colonoscope (or even the same, substantially refurbished instrument) would have required a separate analysis, given that another instrument would have had different native stiffness and flexibility characteristics from the one tested up to this point. The consequent shortfall in sample size means that the power of the statistical equations comparing colonoscopy rates and times between the different interventions has been compromised and the results of those comparisons reported herein should therefore be interpreted with caution. However, the absence of any trend towards statistical significance, manifested by the high P-values for the per-protocol cecal intubation rate and time comparisons, offers some comfort that the finding of no statistically significant difference between these outcomes by intervention is valid. Further support for this conclusion lies in the finding of persistently high P-values after multiple imputation of data (treated as missing) that would have been collected had the planned sample size been accrued. This is an elegant procedure for predicting the likelihood of futility early on in a large trial [14] by projecting (imputing) data based on trends in the existing data.

The validity of the analysis of the effect of application of the stiffening wires after failure of the unaided colonoscope in intervention group A was not predicated on the overall sample size calculation for the primary comparisons. Although the power of this secondary analysis would have improved with a larger sample size in intervention group A, it is reasonable to conclude that the statistically significant improvement in cecal intubation rate detected using the McNemar test for small samples with paired data is valid. It is plausible that over time, the investigator could have become more adept at manoeuvring the unaided colonoscope into the cecum, thereby reducing the failure rate and the need for intervention with the stiffening wires and resulting in a reduction of the statistical significance of the intervention. Were this to occur with accrual of the target sample size, the intervention would nevertheless have been clinically important, the stiffening wires having enabled a complete colonoscopic examination in a substantial number of patients.

The allocation concealment procedure in this study was inadequate as the allocation for the next case was printed on the same page of the randomization schedule as the current allocation and would therefore be visible to the researcher. Foreknowledge of the next allocation theoretically offers the researcher an opportunity to sequence procedures for individual patients so that they receive an intervention which the researcher believes might be more effective in that patient, thereby introducing bias. In practice, this would be very difficult if not impossible to organize since the randomization schedule cannot be altered nor, if a patient is scheduled out of sequence, can the researcher predict that sufficient cases will be recruited in time to close gaps in the schedule. A more reliable concealment procedure, which would also have achieved concealment from the nurses, would have been to seal individual allocations in separate, numbered envelops. Nevertheless, the integrity of the process was maintained by the nurse assistants who affirmed on each occasion that the procedure allotted according to the randomization schedule was the one being performed. In no case was the sequencing of cases (most booked at least 2 weeks in advance) influenced nor changed on the basis of foreknowledge of the next allocation by either the nurses or the researcher.

## Conclusions

Routine application of either wire on entry into the transverse colon neither reduces cecal intubation time nor improves cecal intubation rate compared to the unaided colonoscope. Indeed, there is a non-significant trend suggesting that early, routine application of the wires may reduce the probability of negotiating the hepatic flexure.

However, application of the stiffening wires after intractable failure of the unaided colonoscope enabled a statistically significant improvement in cecal intubation rate. The wires are therefore most effectively applied only after failure of the colonoscope to progress (at any point distal to the splenic flexure).

## Availability of supporting data

The data set supporting the results of this article is available in the LabArchives repository, DOI: http://10.6070/H4V40S4W and hyperlink: https://mynotebook.labarchives.com/share/Jeffrey%2520East's%2520Notebook/MTkuNXw3MzA2LzE1L1RyZWVOb2RlLzkxOTgwMjU0Nnw0OS41.

## Competing interests

The author declares that he has no competing interests. The research and publication of the manuscript were funded entirely by the author from personal resources.

## References

[B1] Ginsberg GG Colonoscopy with the variable stiffness colonoscope Gastrointest Endosc 2003 58 579 584 10.1067/S0016-5107(03)01873-X 14520299

[B2] Baron TH The variable stiffness colonoscope: a scope for all seasons? Am J Gastroenterol 2002 97 2942 2943 10.1111/j.1572-0241.2002.07091.x 12492173

[B3] Shah SG Saunders BP Aids to insertion: magnetic imaging, variable stiffness, and overtubes Gastrointest Endosc Clin N Am 2005 15 673 686 10.1016/j.giec.2005.08.011 16278132

[B4] Sullivan MJ Variable stiffening device for colonoscopy Gastrointest Endosc 1990 36 642 643 10.1016/S0016-5107(90)71208-4 2279681

[B5] Sivak MV Gastroenterologic Endoscopy 1999 Saunders, W.B

[B6] Ruffolo TA Lehman GA Rex D Colonoscope damage from internal straightener use Gastrointest Endosc 1991 37 107 108 2004675 10.1016/s0016-5107(91)70648-2

[B7] Zutron Medical Colonoscope stiffening device 2009 http://zutronmedical.com/endoscope-stiffening-system.php

[B8] Lee SK Kim TI Shin SJ Kim BC Kim WH Impact of prior abdominal or pelvic surgery on colonoscopy outcomes J Clin Gastroenterol 2006 40 711 716 10.1097/00004836-200609000-00010 16940884

[B9] Dupont WD Plummer WD Jr Power and sample size calculations. A review and computer program Control Clin Trials 1990 11 116 128 10.1016/0197-2456(90)90005-M 2161310

[B10] Fisher RA Yates F Smith PG, Morrow RH Appendix 7.1 Field Trials of Health Interventions in Developing Countries: A Toolbox 1996 2 Macmillan Education, London 145 148

[B11] East JM Data from stiffening wire and colonoscopy study. LabArchives 2012 https://mynotebook.labarchives.com/share/Jeffrey%2520East's%2520Notebook/MTkuNXw3MzA2LzE1L1RyZWVOb2RlLzkxOTgwMjU0Nnw0OS41

[B12] Anderson JC Messina CR Cohn W Gottfried E Ingber S Bernstein G Coman E Polito J Factors predictive of difficult colonoscopy Gastrointest Endosc 2001 54 558 562 10.1067/mge.2001.118950 11677470

[B13] Takahashi Y Tanaka H Kinjo M Sakumoto K Prospective evaluation of factors predicting difficulty and pain during sedation-free colonoscopy Dis Colon Rectum 2005 48 1295 1300 10.1007/s10350-004-0940-1 15793639

[B14] Betensky RA Multiple imputation for early stopping of a complex clinical trial Biometrics 1998 54 229 242 10.2307/2534010 9544518

[B15] Odori T Goto H Arisawa T Niwa Y Ohmiya N Hayakawa T Clinical results and development of variable-stiffness video colonoscopes Endoscopy 2001 33 65 69 10.1055/s-2001-11174 11204990

[B16] Othman MO Bradley AG Choudhary A Hoffman RM Roy PK Variable stiffness colonoscope versus regular adult colonoscope: meta-analysis of randomized controlled trials Endoscopy 2009 41 17 24 10.1055/s-0028-1103488 19160154

[B17] Hsieh YH Zhou AL Lin HJ Comparing different methods of activating the variable stiffness function of a pediatric variable stiffness colonoscope J Chin Med Assoc 2008 71 23 29 10.1016/S1726-4901(08)70068-6 18218556

